# Quantitative Shotgun Proteomics Analysis of Rice Anther Proteins after Exposure to High Temperature

**DOI:** 10.1155/2015/238704

**Published:** 2015-11-05

**Authors:** Mijeong Kim, Hijin Kim, Wondo Lee, Yoonjung Lee, Soon-Wook Kwon, Joohyun Lee

**Affiliations:** ^1^Department of Applied Bioscience, Konkuk University, Seoul 143-701, Republic of Korea; ^2^Department of Plant Bioscience, Pusan National University, Milyang 627-706, Republic of Korea

## Abstract

In rice, the stage of development most sensitive to high temperature stress is flowering, and exposure at this stage can result in spikelet sterility, thereby leading to significant yield losses. In this study, protein expression patterns of rice anthers from Dianxi4, a high temperature tolerant Japonica rice variety, were compared between samples exposed to high temperature and those grown in natural field conditions in Korea. Shotgun proteomics analysis of three replicate control and high-temperature-treated samples identified 3,266 nonredundant rice anther proteins (false discovery rate < 0.01). We found that high levels of ATP synthase, cupin domain-containing proteins, and pollen allergen proteins were present in rice anthers. Comparative analyses of 1,944 reproducibly expressed proteins identified 139 differentially expressed proteins, with 95 increased and 44 decreased in response to high temperature conditions. Heat shock, DnaK family, and chaperone proteins showed highly increased expression, suggesting that the high temperature tolerance of Dianxi4 is achieved by stabilization of proteins in pollen cells. Trehalose synthase was also highly increased after heat treatment, suggesting a possible role for trehalose in preventing protein denaturation through desiccation.

## 1. Introduction

Rice is one of the most important staple cereal crops in the world, along with wheat and corn. It is cultivated and consumed mainly in tropical and temperate regions, especially Asia. Cereal crops, such as rice, wheat, and corn, are particularly susceptible to changes in environmental conditions. The International Panel on Climate Change (IPCC) predicts that average global temperatures will increase by 1–4°C by 2100, and this global warming will cause the yields of major crops to decrease by more than 25% by 2050 [1].

Previous studies have described rice yield reductions of 7-8% for each 1°C increase in temperature [2]. Moreover, rice kernel dimensions (length, width, and thickness) decrease at day/night temperatures of 39/34°C [3]. High temperature stress occurring when rice plants are at the developmental stages from booting to flowering is the most influential factor affecting rice yields. High temperatures lead to rice spikelet sterility at the flowering stage due to a reduced number of pollinations on the stigma as a result of obstruction to pollen shedding and a decline in the growth and development of pollen grains [4]. After high temperature treatment for one or two days at the flowering stage, the fertilization ability of rice pollen grains decreases due to inhibition of thecae dehiscence [5], and less than 50% of pollen grains germinate on stigmas under high temperature conditions [6, 7].

In rice, high temperature stress leading to significant yield loss due to spikelet sterility has been studied in various ways. Since resistance to high temperature is a quantitative trait, mapping quantitative trait locus (QTL) using SNP markers is fundamental to the study of this characteristic. QTLs that conferred protection from heat stress have been detected in the tolerant N 22 variety on chromosome 4 (qHTSF4.1) and chromosome 1 (qHTSF1.1), explaining 12.6% and 17.6% of the total phenotypic variation, respectively [8]. Genome expression studies of the molecular response of rice pollen to high temperature conditions have also been reported. RNA microarray analysis identified 1,439 genes as differentially expressed at high temperatures [9]. Fifteen of the genes identified were further analyzed by real-time reverse transcription-PCR and 13 were repressed in response to high temperature. Genes repressed at high temperature were expressed specifically in the immature anther, mainly the tapetum, suggesting an important role for the tapetum in pollen development under high temperature stress [9]. Recently, a miRNA expression study of panicles of the heat tolerant rice cultivar, N 22, under high temperature conditions was reported. A total of 294 known miRNAs and 539 novel miRNAs were identified from young panicles. Among identified miRNAs, 47 were differentially expressed (21 upregulated and 26 downregulated) under heat stress [10]. Gene ontology (GO) category analysis of genes targeted by these miRNAs revealed that these were related to various biological process categories and that, among these, the category of cell growth was enriched. Rice proteome expression patterns in response to heat have also been monitored using 2D-PAGE [11]. Proteome expression patterns were compared among three rice varieties, including a Japonica variety, Moroverekan, which is highly sensitive to high temperature, an Indica variety, IR64, which is adequately tolerant to high temperature, and an Aus variety, N22, which is highly tolerant to high temperature. 2D electrophoresis at pH 4–7 identified 46 differentially expressed proteins. Of these, 13 were analyzed by MALDI-TOF and seven were identified as putative cold shock proteins (CSPs), an inorganic pyrophosphatase, a serine protease (AIR3), a dirigent-like protein, a ribosomal protein (S19), a small heat shock protein (sHSP), an iron deficiency protein (IDS3), and six unknown or hypothetical proteins.

Multidimensional protein identification technology (MudPIT), a representative shotgun proteomic method, has been introduced as a complimentary technique to 2D-PAGE [12]. Also, shotgun proteomics approaches make large scale, high-throughput protein identification possible. The shotgun proteomic method involves separation of trypsin-digested proteins by liquid chromatography, followed by identification by tandem mass spectrometry and interrogation of protein databases [12, 13]. MudPIT allows qualitative, rather than quantitative, analysis; however, relative quantitative analysis is possible by calculating normalized spectral counts [14].

The tiny size of rice pollen grains makes it almost impossible to collect pure samples to monitor proteome responses. Thus, rice anthers are usually collected for protein extraction. Here, we describe quantitative shotgun proteomics analysis, which allowed us to screen a large number of proteins using only a small amount of material from rice anthers. We used this method to compare protein expression patterns in Dianxi4.

## 2. Materials and Methods

### 2.1. Plant Material and High Temperature Treatment

The method of Ye et al. (2012) was modified for heat treatment in flowering stage. Dianxi4, a Chinese rice variety, and Ilpum, a Korean commercial variety, were grown at Konkuk University in an experimental rice paddy field in 15 × 30 cm rows. One day before anthesis, each individual plant including soil was scooped from paddy field with 15 cm diameter and put into a pot. Rice plants were put in a growth chamber at 06:30 AM. Temperature was gradually increased from 27°C to 38°C until 08:00 AM. At 18:30 PM, the temperature was gradually decreased to 24°C until 20:00 PM. The relative humidity was 70% with a 12 h day/12 h night cycle. After 5 days of high temperature treatment, plants were moved to a greenhouse and cultivated until the grain fully matured. As a control, plants with tillers at the same stage of development (bearing panicles) were scooped the same as in the method of the high-temperature-treated rice. The rice in a pot was placed in the rice filed by the end of the day of high temperature treatment; then the control plants were moved to greenhouse with the high-temperature-treated rice plants. Three individual plants for each variety were used and the spikelet fertility was measured from 9 panicles (three panicles per an individual plant). For the proteomic analysis, plants grown in paddy field were put into pots as descried previously. Rice plants were put in a growth chamber at 06:30 AM. Temperature was gradually increased from 27°C to 38°C until 08:00; then the temperature was maintained through day and night. At the next day at 05:00 AM, the temperature was gradually decreased to 24°C until 06:30 AM. The relative humidity was 70% with a 12 h day/12 h night cycle. For the control plants, the rice in a pot was placed in the rice filed. Three individual plants for each variety were used and rice anther for proteome analysis was harvested from one panicle for each plant.

### 2.2. Rice Anther Protein Extraction

Anthers of Dianxi4 were collected immediately after one day of high temperature treatment. To ensure that all anthers collected were at the same developmental stage, they were taken from upper part of only one panicle; anthers emerging from the spikelet were not used in this study. Only anthers inside in the spikelet and located in the middle of panicles approximately 3 cm in width were collected. Control anthers were collected on the same day using the same method. Harvested anthers were ground in liquid nitrogen using metal balls and protein was extracted from the resultant powder using extraction buffer (100 mM Tris-HCl, pH 8.5; 8 M Urea; 5 mM DTT; 1% LDS). The suspension was incubated at room temperature for 30 min, followed by centrifugation at 14,000 g for 15 min. The supernatant was retained and filtered through 0.45 *μ*m membrane filters (Millipore, Billerica, MA, USA). Protein concentration was assayed using the 2D-Protein Quant Kit (GE Healthcare, Piscataway, NJ, USA).

### 2.3. 1D LDS-PAGE and In-Gel Digestion with Trypsin

Protein samples (50 *μ*g) were loaded on to 4–12% Bis-Tris acrylamide gradient gels (Invitrogen, Carlsbad, CA, USA) and stained using a Colloidal Blue Staining Kit (Invitrogen, Carlsbad, CA, USA). Each lane containing separated proteins was sliced into seven pieces, which were then cut into smaller cubes (approximate size, 1 mm^3^) and transferred to Eppendorf tubes. Sliced gels were destained with destaining buffer (50 mM NH_4_HCO_3_, pH 7.8 in 50% acetonitrile) and dehydrated in a SpeedVac. Dried samples were reduced at 56°C for 45 min in 10 mM DTT (in 25 mM NH_4_HCO_3_) and alkylated with alkylation buffer (55 mM iodoacetamide in 25 mM NH_4_HCO_3_) for 30 min at 24°C in a dark place. Reduced and alkylated samples were dried in a SpeedVac, mixed with digestion buffer (12.5 ng/*μ*L trypsin in 50 mM NH_4_HCO_3_), and incubated at 36°C overnight. Tryptic peptides were harvested from gel slices using harvest buffer (5% formic acid in 50% acetonitrile) and incubated at RT for 20 min. Combined supernatants were dried out in a SpeedVac, desalted using Pierce C18 spin columns (Thermo Scientific, Rockford, IL, USA), and analyzed by LC MS/MS.

### 2.4. LC MS/MS Analysis with Q Exactive

A nanoflow HPLC instrument (Easy nLC, Thermo Fisher Scientific, San Jose, CA, USA) coupled on-line to a Q Exactive mass spectrometer (Thermo Fisher Scientific, Bremen, Germany) was used. Analytical columns (12 cm; 75 *μ*m inner diameter) were packed in-house with Alltima C18-AQ 5 *μ*m resin. Samples were separated with a linear gradient of 3–60% buffer B at a flow rate of 250 nL/min. The total run time for an LC MS/MS was 110 min. MS data was acquired using the data-dependent top8 method, dynamically choosing the most abundant precursor ions from the survey scan (300–2,000 Da) for higher-energy collisional dissociation (HCD) fragmentation. The dynamic exclusion duration was 60 s, and the isolation window for precursors was set to 4* m/z*. Survey scans were acquired at a resolution of 70,000 at* m/z* 200 and the resolution for HCD spectra was set to 17,500 at* m/z* 200.

### 2.5. Protein Identification and Comparative Analysis of Relative Protein Abundance

Each peptide from the LC-MS/MS spectra was searched against the TIGR Rice Pseudomolecule protein database Release V7.0 (http://rice.plantbiology.msu.edu/annotation_pseudo_current.shtml) using Proteome Discoverer (Thermo Fisher Scientific, version 1.3) software. False discovery rate (FDR) in peptide identification through spectrum match to peptide was evaluated by using a decoy database which was created by reversing all the rice protein sequences and it was set to a maximum of 0.01. For the peptides shared between multiple proteins, identified proteins were grouped by the algorithm implemented in Proteome Discoverers (Thermo Fisher Scientific, version 1.3) and the spectral counts (number of peptide spectrum matches) for proteins include those redundantly identified. Carbamidomethylation of cysteine was set as a field modification and oxidation of methionine was set as a variable modification for database searching.

The output of the Proteome Discoverer search was exported to Microsoft Excel to calculate normalized spectral counts (NSpC) [15–17]. The NSpC for each protein *k* is given by

(1) $$\begin{array}{c} {\left(\;\text{NSpC}\;\right)}_{k}=\frac{{\left(\text{SpC}/L\right)}_{k}}{\sum_{i=1}^{n}{\left(\text{SpC}/L\right)}_{i}}, \end{array}$$

where the whole number of MS/MS spectra matching peptides from protein *k* (SpC) is divided by the protein's length (*L*), then by SpC/*L* for all (*N*) proteins in the experiment.

### 2.6. Bioinformatics Analysis of Proteomics Data

Rice protein molecular weights (MW) and *pI* values were calculated using EMBOSS Pepstats, EMBL-EBI (http://emboss.sourceforge.net/download/), and the EMBOSS Pepstats algorithm (http://emboss.bioinformatics.nl/cgi-bin/emboss/pepstats).

GO annotations of rice proteins were retrieved from the TIGR Rice Pseudomolecule protein database; Release V7.0. GO singular enrichment analyses were performed in agriGO (http://bioinfo.cau.edu.cn/agriGO/analysis.php) [18].

## 3. Results and Discussion

### 3.1. Heat Temperature Tolerance of Dianxi4

**Figure 1:**
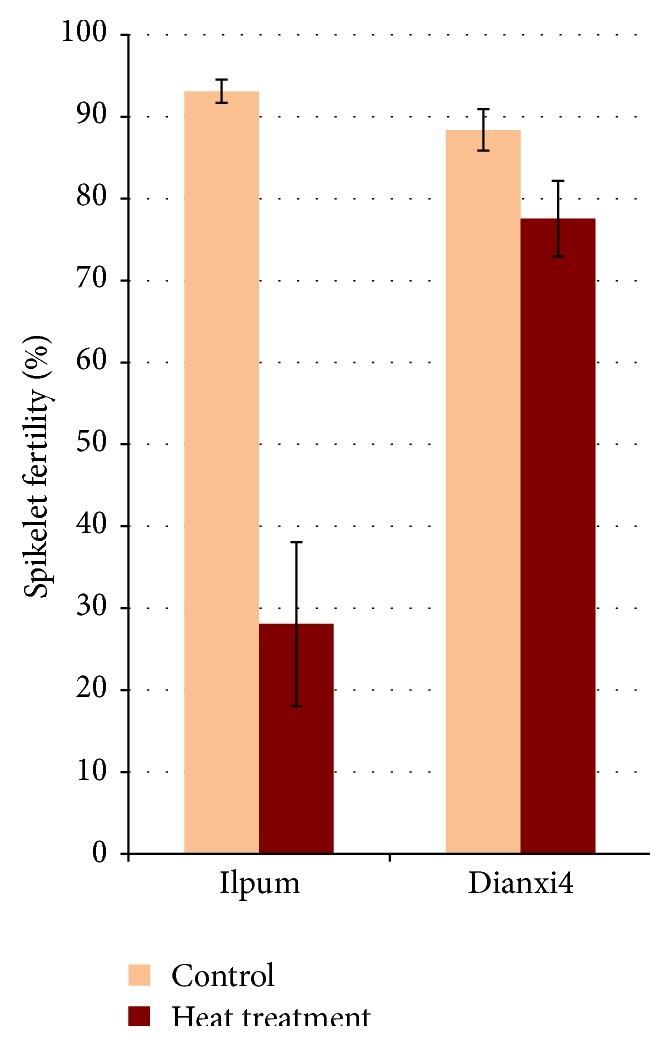
Comparison of spikelet fertility of Ilpum and Dianxi4 at (orange) normal condition and (brown) heat stress.

Ilpum, a commercial rice variety, was included in our initial experiments to compare the tolerance of the Dianxi4. One of the most heat-sensitive stages of rice pollen development is flowering; thus, high temperature was applied to the plants at the flowering stage. After 5-day heat-treatment both rice varieties were cultivated in a greenhouse and spikelet fertility was measured after the grain reached full maturity (Figure 1). For Ilpum, fertility was 28.1 ± 10.0 after high temperature treatment and 93.1 ± 1.4 under normal conditions, indicating that the high temperature treatment was effective. For Dianxi4, fertility under normal conditions was 88.4 ± 2.5 and, even after high temperature treatment, fertility was maintained at 77.6 ± 4.6. Dianxi4 showed high temperature tolerance in the flowering stage.

### 3.2. Identification of Proteins in Anthers of Dianxi4 Rice at the Flowering Stage

**Figure 2:**
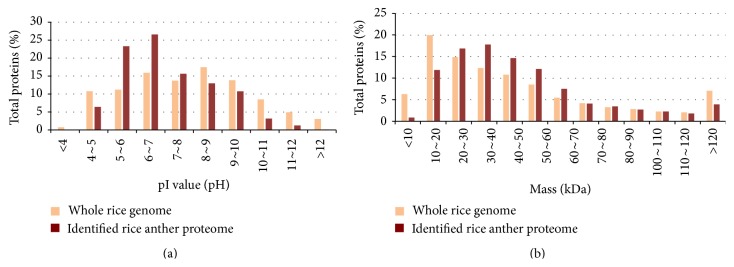
Distribution *pI* value and molecular weight of proteins extracted from (brown) Dianxi4 anthers and those of proteins encoded by the whole rice genome.

Proteins from anthers of Dianxi4 rice plants exposed to high temperature treatment and grown under normal conditions were identified by shotgun proteomic analysis; anthers were used rather than pollen due to the small size of rice pollen grains. Individual anthers are also too small to provide sufficient proteins for proteomic analysis; therefore, groups of anthers were collected from single panicles and the pooled proteins extracted. For biological replication, anthers were collected from different individual plants. Shotgun proteomics analysis of three replicates each of the control and high-temperature-treated samples identified 3,266 nonredundant proteins (Supplementary Table 1; see Supplementary Material available online at http://dx.doi.org/10.1155/2015/238704). The number of identified proteins common to all six samples was approximately 2,000 (Supplementary Table 2), indicating that not all 3,266 proteins were identified in each sample. This is likely due to the phenomenon of “analytical incompleteness,” where any single analytical run during shotgun proteomic analysis may identify only a fraction of relevant peptides in a very highly complex mixture [19]. The distribution of the physiochemical characteristics of the identified 3,266 nonredundant proteins were compared with those encoded by the entire rice genome (TIGR Rice Pseudomolecule protein database Release V7.0) (Figure 2). The *pI* values of the identified proteins ranged from 3.93 (LOC_Os07g41694.1) to 12.48 (LOC_Os01g69020.1). The proportion of identified proteins with *pI* values of 5–7 was relatively large compared with those encoded by the whole genome; however, the *pI* values of the identified proteins were dispersed across a wide range. The MW of the identified proteins also had a broad range, from 5.29 kDa (LOC_Os04g02670.1) to 486 kDa (LOC_Os09g07300.1), although the proportion of proteins of < 20 kDa was below that of all rice proteins. This result is due to the fact that low MW proteins produce fewer peptides that can be detected in a mass spectrometer after digestion with trypsin.

Relative protein quantities can be estimated from the normalized Spectral Count with a high degree of accuracy. In general, proteome studies of rice leaves have found RuBisCO protein and photosynthesis proteins to be present in large quantities. Interestingly, our analysis of total proteins identified from rice anthers revealed that pollen allergen proteins (LOC_OS06g451810, LOC_OS09g23899, LOC_OS09g23999, and LOC_OS04g25190), ATP synthase, and cupin domain-containing proteins were present in large quantities. Pollen allergen proteins are allergenic to humans but are present in high amounts in plants, suggesting that they have physiological roles required for pollen germination and growth [20]. Our proteomic analysis confirms that high quantities of these proteins are present in rice anthers. The observed high levels of ATP synthase (LOC_OS01g49190) suggest a role for this protein in the energy-requiring process of pollen tube germination. Two cupin domain-containing proteins (LOC_OS05g0250 and LOC_OS01g74480) were also present at high levels. Previous studies show that cupins belong to some 18 different functional classes and were originally detected during the early phase of germination in wheat embryos [21]. Given the high amounts detected in rice anthers, and the previously reported roles of pollen allergens, cupin, and ATP synthase, the results presented herein suggest possible function roles for these proteins in pollen germination.

### 3.3. Proteome Expression Patterns after High Temperature Treatment

**Figure 3:**
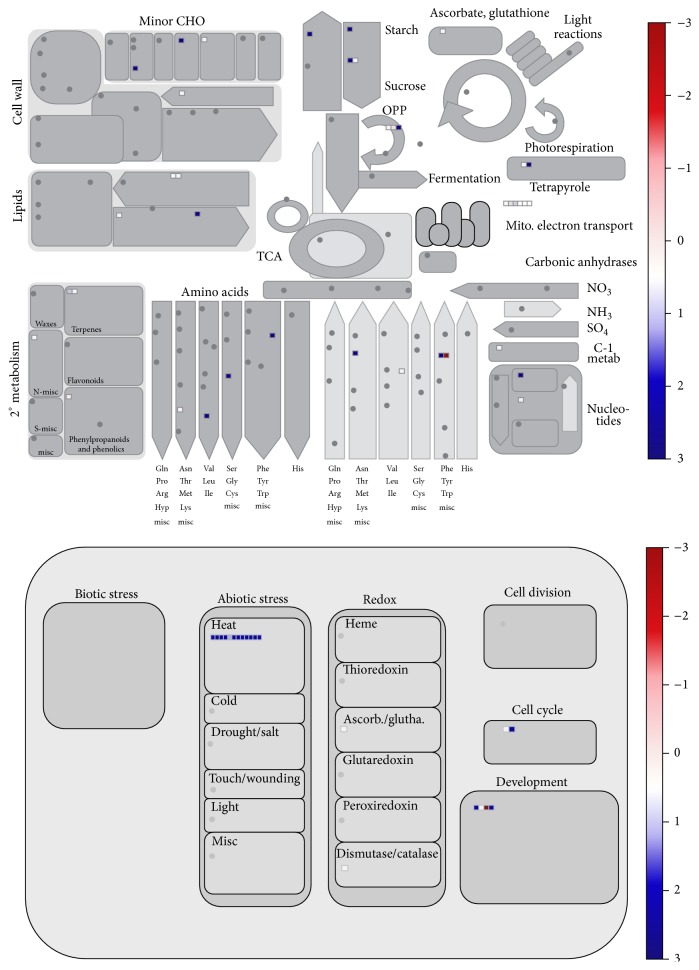
Mapman analysis for expression pattern of the differentially expressed proteins responding to heat stress.

For comparative analysis, the relative quantities of the identified proteins were calculated using the label-free spectral count (SC) method. Not all 3,266 identified proteins were reproducibly identified in all control or heat-treated samples; therefore, we included only proteins identified in all three replicates of either category, with at least two SCs per replicate. Using these criteria, SCs of 1,944 reproducible proteins were normalized (NSpC) and the logarithmically transformed NSpCs (the natural log (Ln) of NSpC) were compared using a *t*-test (Supplementary Table 3). The average coefficient of determination (*R*2) between NSpCs for the biological replicates was 0.84. Proteome changes were detected using a *t*-test (*α* = 0.05) to compare the expression patterns of Dianxi4 grown under normal conditions with those after exposure to high temperature. A total of 139 proteins were differentially expressed between control and treated samples (Supplementary Table 4). Among these 139 proteins, 95 showed increased and 44 decreased expression after high temperature treatment. All 139 differentially expressed proteins were analyzed by GO category enrichment analysis to determine enriched GO terms in biological processes, cellular components, or molecular functions categories [22]. Interestingly, in the response to high temperature, one molecular function and 19 cellular component GO terms were identified (Table 1). The fact that the majority of the enriched GO terms were cellular components indicates that the response to high temperature stress affects proteins associated with various cellular components. Considering that the damage caused to pollen by high temperature stress results in the inability of pollen to be successfully germinated, the dynamic changes in protein expression associated with cellular components (representing changes in expression levels of these proteins) may be not only a consequence of damage resulting from high temperature stress, but also the result of the tolerance response required to maintain proper cellular components for recovery. Next, we conducted Mapman analyses [23] to identify changes in metabolic pathways and other processes (Figure 3). Some metabolic pathways were increased after heat treatment, including lipid, starch, and some amino acid metabolic pathways. Interestingly, the heat response process was also increased; among the 12 proteins involved in the heat response, nine were heat shock proteins (HSP), two were DnaK family proteins, and one was a chaperone protein.

### 3.4. Differentially Expressed Proteins in Rice Anther after High Temperature Treatment

Among the 96 proteins showing increased expression in heat-treated anthers relative to control anthers, nine were HSPs, and all were expressed at high levels (Table 2). Several rice proteomic studies report upregulation of HSPs in response to high temperatures [11, 24, 25]. Together, these previous studies and our results strongly suggest that HSPs are associated with improved tolerance to high temperature stress. HSPs perform molecular chaperone activities, contributing to cellular homeostasis in cells under both normal and adverse growth conditions [26] and can be divided into high (HSP100, HSP90, HSP70, and HSP60) and small molecular mass proteins (HSP20 or small heat shock proteins (sHSPs)) [27]. Interestingly, there were seven sHSPs among the nine HSPs showing increased expression after heat treatment. A previous study by Chen et al. also detected upregulation of several sHsps in rice anthers under high temperature conditions [28]. The results of this proteomics study confirm a potentially important role for sHSPs in rice anthers subjected to high temperature stress; therefore, we conclude that upregulation of sHSPs is likely to be important in protecting rice pollen from the adverse effects of high temperature exposure. In addition, four proteins functionally associated with the stabilization of protein structure, one chaperone protein, two DnaK family proteins, and one late embryogenesis abundant protein, were upregulated after high temperature treatment. Thus, the proteins upregulated in rice anthers in response to heat stress are required for maintenance of cellular homeostasis through the stabilization of proteins required for pollen germination. In addition, three glutathione S-transferases and one superoxide dismutase protein were increased after heat shock treatment. These proteins are generally expressed under various stress conditions [29, 30]. Although one of the glutathione S-transferases demonstrated highly increased expression, the overall responses of these antioxidant proteins in the rice anther were less pronounced than those of HSPs and other proteins with chaperone functions. Furthermore, one trehalose synthase protein demonstrated highly increased expression in response to heat treatment. Trehalose is a natural alpha-linked disaccharide formed by a *α*,*α*-1,1-glucoside bond between two *α*-glucose units and is regarded as a protective metabolite against various stress conditions during exposure to different environmental stresses [31]. Although the molecular mechanism underlying trehalose protection is not yet fully understood, one hypothesis proposes that, especially during desiccation and heat stress, trehalose replaces water and binds via hydrogen bridges to polar protein residues to prevent the denaturation of protein molecules [31]. The proposed role of trehalose is consistent with that of HSPs, which were also found to be increased in response to heat-treatment in this study. In addition to those proteins with previously reported roles, eight of unknown functions showed increased expression in response to heat treatment (descriptions of these proteins are provided in the rice genomic database; TIGR Rice Pseudomolecule protein database, Release V7.0). Due to our experimental design, it is not clear whether upregulation of these proteins is associated with high temperature tolerance or a consequence of stress induced damage; thus, further studies are required to confirm their roles.

Of the proteins showing decreased expression after heat treatment, the majority showed minimal decreases in expression (Supplementary Table 3). The protein showing the greatest reduction in expression was LOC_Os03g15320.1 (glyoxal oxidase-related), which is an extracellular H_2_O_2_-producing enzyme; its decrease is likely associated with upregulation of antioxidant proteins; however, its exact role in response to high temperature is not clear. The previously reported functions of the other downregulated proteins were metabolism and catalytic activity, suggesting that high temperature stress may affect certain metabolic processes.

## 4. Conclusion

In summary, through quantitative shotgun proteomic analysis of rice anthers, we identified 3,266 nonredundant rice proteins, one of the largest rice anther protein identification experiments to date. ATP synthase, cupin domain-containing proteins, and pollen allergens were present in the rice anther in large amounts, suggesting a potentially important physiological role for rice pollen allergens. A comparison of protein expression patterns in rice anthers cultivated under normal conditions or exposed to high temperature treatment at the flowering stage suggested that the tolerance of Dianxi4 may be due to highly increased expression of heat shock, DnaK family, chaperone, and trehalose synthase proteins, which maintain protein homeostasis in pollen cells in response to high temperatures.

## Supplementary Material

Supplementary Table 1: List of total 3,266 identified non-redundant proteins.
Supplementary Table 2: Raw files for the identified proteins including detected peptides.
Supplementary Table 3: Relative expression amount of the 1,944 proteins.
Supplementary Table 4: Differentially expressed proteins.

## Figures and Tables

**Table 1:** Enriched GO terms of the differentially expressed proteins in Dianxi4 anther at high temperature.

GO term	Ontology^a^	Description	Number in input list	Number in rice genome	*p* value	FDR
GO:0003824	F	Catalytic activity	71	13508	5.80*E* − 04	1.50*E* − 02
GO:0005737	C	Cytoplasm	87	11866	2.70*E* − 13	3.80*E* − 11
GO:0044424	C	Intracellular part	94	14514	2.00*E* − 11	1.40*E* − 09
GO:0044444	C	Cytoplasmic part	79	10930	4.00*E* − 11	1.90*E* − 09
GO:0005622	C	Intracellular	94	15144	3.20*E* − 10	1.10*E* − 08
GO:0005739	C	Mitochondrion	22	1611	2.70*E* − 07	7.60*E* − 06
GO:0044464	C	Cell part	103	19532	3.30*E* − 07	7.80*E* − 06
GO:0043229	C	Intracellular organelle	74	12251	1.60*E* − 06	2.80*E* − 05
GO:0043226	C	Organelle	74	12251	1.60*E* − 06	2.80*E* − 05
GO:0005623	C	Cell	109	22048	3.10*E* − 06	4.80*E* − 05
GO:0043227	C	Membrane-bounded organelle	71	11878	5.30*E* − 06	6.70*E* − 05
GO:0043231	C	Intracellular membrane-bounded organelle	71	11878	5.30*E* − 06	6.70*E* − 05
GO:0009536	C	Plastid	35	4703	7.70*E* − 05	9.00*E* − 04
GO:0005829	C	Cytosol	27	3345	1.70*E* − 04	1.90*E* − 03
GO:0005618	C	Cell wall	14	1179	2.00*E* − 04	2.00*E* − 03
GO:0030312	C	External encapsulating structure	14	1189	2.20*E* − 04	2.10*E* − 03
GO:0005773	C	Vacuole	17	1723	3.70*E* − 04	3.20*E* − 03
GO:0043232	C	Intracellular non-membrane-bounded organelle	13	1237	1.10*E* − 03	8.30*E* − 03
GO:0043228	C	Non-membrane-bounded organelle	13	1237	1.10*E* − 03	8.30*E* − 03
GO:0005730	C	Nucleolus	7	495	3.30*E* − 03	0.024

^a^P: biological process; C: cellular component; F: molecular function.

**Table 2:** Upregulated rice anther protein against heat stress.

Accession	Description	Expression ratio (treatment/control)
LOC_Os02g52150.2 protein	Heat shock 22 kDa protein, mitochondrial precursor, putative, expressed	57.21
LOC_Os05g44340.1 protein	Heat shock protein 101, putative, expressed	471.87
LOC_Os03g18200.2 protein	Heat shock protein DnaJ, putative, expressed	27.42
LOC_Os03g15960.1 protein	HSP20/alpha crystallin family protein, putative, expressed	104.49
LOC_Os03g16030.1 protein	HSP20/alpha crystallin family protein, putative, expressed	96.61
LOC_Os03g16040.1 protein	HSP20/alpha crystallin family protein, putative, expressed	96.13
LOC_Os01g08860.1 protein	HSP20/alpha crystallin family protein, putative, expressed	54.86
LOC_Os04g36750.1 protein	HSP20/alpha crystallin family protein, putative, expressed	53.11
LOC_Os03g14180.1 protein	HSP20/alpha crystallin family protein, putative, expressed	51.85
LOC_Os02g08490.1 protein	Chaperone protein clpB 1, putative, expressed	2.60
LOC_Os03g16920.1 protein	DnaK family protein, putative, expressed	774.59
LOC_Os05g35400.1 protein	DnaK family protein, putative, expressed	262.28
LOC_Os07g17120.1 protein	Late embryogenesis abundant protein, putative, expressed	32.79
LOC_Os05g02530.1 protein	Glutathione S-transferase, N-terminal domain-containing protein, expressed	1.47
LOC_Os07g28480.1 protein	Glutathione S-transferase, putative, expressed	19.70
LOC_Os01g55830.1 protein	Glutathione S-transferase, putative, expressed	1.43
LOC_Os05g25850.1 protein	Superoxide dismutase, mitochondrial precursor, putative, expressed	1.30
LOC_Os01g53000.1 protein	Trehalose synthase, putative, expressed	78.82
